# Fatal outcome after brain stem infarction related to bilateral vertebral artery occlusion - case report of a detrimental complication of cervical spine trauma

**DOI:** 10.1186/1754-9493-5-18

**Published:** 2011-07-14

**Authors:** Hiroyuki Yoshihara, Todd F VanderHeiden, Yasuaki Harasaki, Kathryn M Beauchamp, Philip F Stahel

**Affiliations:** 1 Department of Orthopaedic Surgery, Denver Health Medical Center, University of Colorado School of Medicine, 777 Bannock Street, Denver, CO 80204, USA; 2 Department of Neurosurgery, Denver Health Medical Center, University of Colorado School of Medicine, 777 Bannock Street, Denver, CO 80204, USA

## Abstract

**Background:**

Vertebral artery injury (VAI) after blunt cervical trauma occurs more frequently than historically believed. The symptoms due to vertebral artery (VA) occlusion usually manifest within the first 24 hours after trauma. Misdiagnosed VAI or delay in diagnosis has been reported to cause acute deterioration of previously conscious and neurologically intact patients.

**Case presentation:**

A 67 year-old male was involved in a motor vehicle crash (MVC) sustaining multiple injuries. Initial evaluation by the emergency medical response team revealed that he was alert, oriented, and neurologically intact. He was transferred to the local hospital where cervical spine computed tomography (CT) revealed several abnormalities. Distraction and subluxation was present at C5-C6 and a comminuted fracture of the left lateral mass of C6 with violation of the transverse foramen was noted. Unavailability of a spine specialist prompted the patient's transfer to an area medical center equipped with spine care capabilities. After arrival, the patient became unresponsive and neurological deficits were noted. His continued deterioration prompted yet another transfer to our Level 1 regional trauma center. A repeat cervical spine CT at our institution revealed significantly worsened subluxation at C5-C6. CT angiogram also revealed complete occlusion of bilateral VA. The following day, a repeat CT of the head revealed brain stem infarction due to bilateral VA occlusion. Shortly following, the patient was diagnosed with brain death and care was withdrawn.

**Conclusion:**

Brain stem infarction secondary to bilateral VA occlusion following cervical spine trauma resulted in fatal outcome. Prompt imaging evaluation is necessary to assess for VAI in cervical trauma cases with facet joint subluxation/dislocation or transverse foramen fracture so that treatment is not delayed. Additionally, multiple transportation events are risk factors for worsening when unstable cervical injuries are present. Close attention to proper immobilization and neck position depending on the mechanism of injury is mandatory.

## Introduction

VAI after blunt cervical trauma had previously been considered to be rare until clinical studies revealed data to the contrary [1,2]. Biffl et al. found blunt VAI in 15.2% of patients who met their screening criteria for digital subtraction angiography [1]. Hyperextension injuries, with or without lateral flexion and rotation, have previously been discussed as the most common mechanism of closed injury to the VA [3,4]. VAI can be unilateral or bilateral. Bilateral VAI is less frequent. Unilateral occlusion of the vertebral artery seldom results in a neurological deficit if the collateral supply through the other vertebral and posterior inferior arteries is sufficient [5]. Bilateral occlusion may also be asymptomatic [4]. However, this usually causes neurological deficit or even death [6]. VAI has been underdiagnosed or misdiagnosed frequently. This mainly occurs because most VAI patients remain asymptomatic if the VA is damaged only unilaterally. However, misdiagnosed VAI has often been reported to cause acute neurologic deterioration of previously conscious patients with cervical spine injury [7-9].

In the present paper, we report a case of a 67 year-old male who sustained a severe hyperextension injury to the C5-6 segment as the result of a roll-over MVA. This fracture-subluxation also caused bilateral VA injury that progressed to brain stem infarction and, ultimately, death.

## Case report

**Figure 1:**
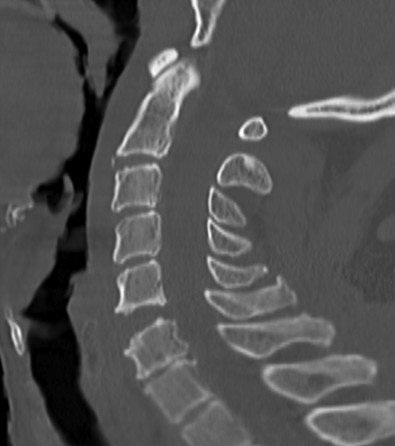
**Sagittal cervical spine CT reconstruction-image at the local hospital shows subluxation at C5-C6 with disk-space diastasis demonstrating concern for extension-distraction injury**.

**Figure 2:**
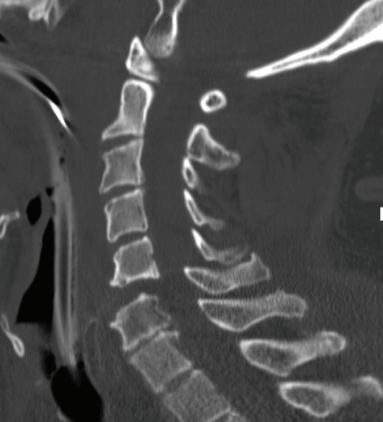
**Sagittal cervical spine CT reconstruction-image at our institution shows dramatically worsened subluxation at C5-C6**.

**Figure 3:**
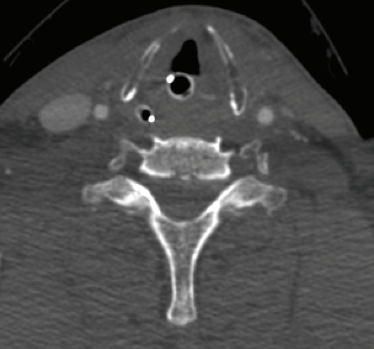
**Neck CT angiogram axial-image shows complete occlusion of bilateral VA**. Note the absence of contrast dye within the foramen transversarium bilaterally whereas bilateral carotid arteries are contrast filled.

**Figure 4:**
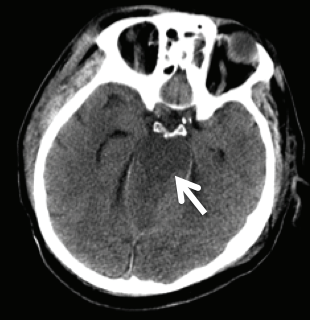
**Cranial CT image obtained on the second hospital day at our institution**. This axial cut demonstrates extensive brain stem infarction (white arrow) attributed to bilateral vertebral artery occlusion.

A 67 year-old male had a single-car, rollover MVC. At the scene, emergency medical response personnel noted that he was alert, oriented and could move all extremities vigorously. No focal neurological deficit was noticed. He complained of facial pain which was attributed to a minor right-temple-area laceration. However, his most significant complaint was posterior neck pain. He was fitted with a cervical field-collar and transported to the local hospital. X-rays taken at that time revealed traumatic C5-C6 retrolisthesis. A cranial CT showed no intracranial abnormality with the absence of bleeding or traumatic injury. A cervical spine CT revealed subluxation at C5-C6 (Figure 1) and a comminuted fracture of the left lateral mass of C6 with disruption of the transverse foramen. Lack of spine treatment capability prompted the patient's transfer to a regional medical center by life flight four hours later. Upon arrival, a neurosurgical evaluation noticed development of neurological deficits. Bilateral upper extremity weakness was described as 4-/5 in the left biceps and 3+/5 in the right biceps. Unfortunately, no other neurological examination details were available. Then, three hours later, the patient's neurological exam drastically changed. Complete absence of motor function in the upper and lower extremities was described. Additionally, he became apneic and hypotensive requiring cardiopulmonary resuscitation. Intubation and a major resuscitative event restored hemodynamic stability and thus enabled another transfer to our Level 1, Regional Trauma Center. Upon arrival to our institution, he was intubated, and found to have asymmetric pupils, minimal corneal reflex, and minimal cough reflex. These findings were consistent with significant brainstem injury. A repeat cranial CT, repeat cervical spine CT, and a head-neck CT angiogram were obtained. These images revealed dramatically worsened subluxation at C5-C6 (Figure 2) compared to initial, outside imaging studies. Further imaging data revealed complete occlusion of bilateral VA (Figure 3), although reconstitution from anterior circulatory flow was present. Filling defects within the reconstituted vertebral arteries and basilar artery were concerning for dissection and/or thrombus. A repeat head CT obtained 24 hours later demonstrated extensive brain stem infarction (Figure 4). The patient was subsequently diagnosed with brain death. With consent from the patient's family, the patient's care was then withdrawn.

## Discussion

VAI after blunt cervical trauma is common. The VA is most susceptible at the C5-C6 level just after it enters the transverse foramen. Other areas of susceptibility are located at the atlanto-axial level just after the VA leaves the transverse foramen, and at the atlanto-occipital level where the VA is tethered by the dura [4]. Ozveren et al. mentioned that the mobility of the artery suddenly decreases as it enters the transverse foramen of C-6. Bilateral VA injury is frequently associated with midcervical subluxations and dislocations, most commonly at C5-C6 [6]. The associated spine injury may be a dislocation or a vertebral body fracture with transverse foramen involvement. Hyperextension injuries, with or without lateral flexion and rotation, have previously been accepted as the most common mechanism of closed injury to the vertebral artery [3,4]. Biffl et al. suggested VAI should be suspected in any patient who presents with hemorrhage from the mouth, nose, or ear and any mechanism of injury significant for severe cervical hyperextension/rotation or hyperflexion [1]. This case involved a severe hyperextension mechanism with resultant subluxation injury at C5-C6 with transverse foramen fracture. These attributes suggests a high risk of bilateral VAI. The mechanism of injury to the vertebral artery by closed trauma involves stretching and tearing of the intima and media, dissections, mural thrombosis, clot propagation, aneurismal dilation, and occlusion [10,11].

VAI requires a high index of suspicion for prompt diagnosis and successful treatment. The difficulty with diagnosis lies in the fact that the majority of patients will not have neurologic symptoms. Unilateral occlusion of the vertebral artery system rarely results in a neurologic deficit because of collateral supply through the contralateral vertebral artery and the posterior inferior cerebellar arteries [5]. Even if the patient ultimately demonstrates neurological symptoms, it is usually not clearly evident from the initial evaluation [5,12]. Many of the reported cases of posttraumatic VA occlusion demonstrate an asymptomatic interlude of a few hours between the trauma and the onset of signs of brain stem or cerebellar dysfunction [3,5,10,12-14]. Marks and Freed suggested that this interlude was due to the slow progression of the thrombosis or, alternatively, to the gradual swelling of the vessel wall secondary to trauma [13]. Another cause might be the slow swelling of the infracted brain stem and cerebellum which are only partially supplied by collaterals.

Unlike unilateral VA occlusion in which only 20% of patients are symptomatic [15], most patients with bilateral VA occlusion are symptomatic. Patients with bilateral VAI usually presented with more severe symptoms, including an altered mental status, pinpoint pupils, and even sudden respiratory arrest [6,12,16,17]. Occasionally, bilateral VAI patients remain asymptomatic [1,2,18]. Six et al reported a case of asymptomatic posttraumatic bilateral vertebral artery occlusion [4]. Angiography demonstrated occlusion of both vertebral arteries and reconstitution by intramuscular collateral vessels of the thyrocervical trunk and by collaterals from the superficial occipital artery. The absence of neurologic deficits has been attributed to adequate intracranial collateral flow from the anterior half of the circle of Willis. However, acute bilateral vertebral injuries are usually associated with vertebrocerebellar stroke and/or death [17]. In a recent review, the mortality rate for cervical spine fractures associated with VAI was 40% [19]. Previous studies report fully oriented, neurologically normal patients upon initial evaluation with a subsequent progression to brain stem infarction. These infarctions are secondary to bilateral VA occlusion [6,12]. In this case, we speculate a gradual VA occlusion occurring within hours that lead to brain stem infarction despite a brief period when adequate blood flow was present. The prognosis of bilateral VAI is grim with less than a third of the patients achieving good outcome [6].

There have been no definite standards or guidelines as to what is the most important and optimal imaging study for patients suspected of VAI [20]. Being that digital subtraction angiography (DSA) is the criterion standard, neither magnetic resonance angiography (MRA) or computed tomography angiography (CTA) has been able to show comparable sensitivity and specificity [2,21,22]. Recent trauma practice guidelines with associated review suggests that multidetector (> = 8) CTA may approach DSA in terms of sensitivity [23]. In terms of practicality, however, DSA is invasive and resource intensive. Giacobetti et al reported MRA can be an excellent, rapid, noninvasive alternative for diagnosis of vascular occlusion or damage, since most patients with cervical spine injuries will undergo MR imaging for the spinal lesions [24]. CTA is also a useful, less-invasive modality in which examination times are short and contrast volumes are small (usually less than for DSA). At some institutions, polytrauma patients receive a multi-slice CT scan with contrast medium as part of a standardized diagnostic procedure. This potentially leads to earlier diagnosis of VAI. Initial use of contrast enhanced CT scans and/or MRI scans may be wise during initial trauma patient evaluation with severe cervical hyperextension/rotation or hyperflexion. This would also eliminate secondary procedures that are normally obtained after initial non-contrast CT scans identify cervical spine injuries that have potential vertebral artery involvement.

Reported treatments for VAI include supportive management, surgical ligation, radiologic embolization, systemic heparinization, and antiplatelet therapy [12]. The most frequently used specific treatment is anticoagulation. Treatment of bilateral VAI is still not standardized and varies from anticoagulation to ligation and thrombectomy. Urgent reduction of spinal subluxation/dislocation and proper immobilization of the spine with subsequent stable internal fixation is performed in most cases of bilateral VAI [6]. There are no reported cases of attempts of revascularization in traumatic bilateral VAI.

Neurological deterioration in patients with spinal cord injury occurs in around 5% of patients even with proper immobilization of the spine [25]. On the other hand, Hauswald et al. suggest that, as a large amount of force is required to damage the spine and injure the spinal cord, movements during transport are unlikely to generate sufficient energy to result in additional injury [26]. In our patient, cervical spine CT at our institution showed dramatically worsened subluxation at C5-C6 compared to that at the local hospital. Onset of spinal cord injury, or worsening of previously present spinal cord injury, from two major transport events may have occurred. This may be contributing to the progressive neurologic deficit and clinical course. Although full evaluation of the neurological status was not possible once the patient arrived at our institution, the remote data would indicate a relatively drastic change in the perceived motor function of this patient. It also remains possible that VAI worsened more rapidly because of increased subluxation in this patient. More displacement could have resulted in further disruption of the VA system. This possibility demonstrates the importance of proper immobilization and good neck positioning. Depending on the mechanism and morphology of cervical spine injury, proper immobilization could come in different forms. It could be argued that an injury of this severity mandates halo-vest placement especially when multiple, long-distance transfers are needed to facilitate patient care. It should further be mentioned that multiple transportation events can be risk factors for worsening injury and significant delays in treatment.

## Conclusion

This patient died from brain stem infarction secondary to bilateral VA occlusion following blunt cervical spine trauma. Multiple transportation events, poor neck positioning, and improper immobilization seemed to result in worsened subluxation at the injured cervical segment. This may have contributed to the rapid decline in this patient's course from both a neurological and vascular standpoint. Prompt treatment of this injury would have required urgent arteriography to identify the occluded vertebral arteries. It may, therefore, be reasonable to include contrast with initial cervical CT scan evaluation. Alternatively, empirical treatment based on recognition of the traumatic mechanism and pattern of injury may have proved useful. However, no current studies address this concept. Finally, multiple transportation events should be avoided in all possible scenarios and stable, proper immobilization with correct neck position depending on the mechanism of injury is necessary. These fundamental principles may enable prevention of secondary injury to the damaged cervical spine and the important vascular structures of this region.

## Consent

Written informed consent was obtained from the patient's relatives for publication of this case report.

## Competing interests

PFS has received speaker's honoraria by Synthes (Paoli, PA) and Stryker Spine (Allendale, NJ). The authors declare no other competing interests related to this case report.

## Authors' contributions

YH and KMB took care of the patient. HY, TV and PFS wrote the manuscript. All authors contributed to the revisions of the text and approved the final version of this manuscript.

## References

[B1] Biffl WL Moore EE Elliott JP Ray C Offner PJ Franciose RJ Brega KE Burch JM The devastating potential of blunt vertebral arterial injuries Ann Surg 2000 231 672 681 10.1097/00000658-200005000-00007 10767788 PMC1421054

[B2] Miller PR Fabian TC Croce MA Cagiannos C Williams JS Vang M Qaisi WG Felker RE Timmons SD Prospective screening for blunt cerebrovascular injuries: analysis of diagnostic modalities and outcomes Ann Surg 2002 236 386 395 10.1097/00000658-200209000-00015 12192325 PMC1422592

[B3] Simeone FA Goldberg HI Thrombosis of the vertebral artery from hyperextension injury to the neck J Neurosurg 1968 29 540 544 10.3171/jns.1968.29.5.0540

[B4] Six EG Stringer WL Cowley AR Davis CH Posttraumatic bilateral vertebral artery occlusion J Neurosurg 1981 54 814 817 10.3171/jns.1981.54.6.0814 7017076

[B5] Golueke P Sclafani S Phillips T Goldstein A Scalea T Duncan A Vertebral artery injury. Diagnosis and management J Trauma 1987 27 856 865 10.1097/00005373-198708000-00003 3612862

[B6] Ozveren F Ziyal IM Bejjani GK Yaymaci Y Turgay Bilge Bilateral vertebral artery occlusion following cervical spine trauma - case report Neurol Med Chir (Tokyo) 1999 39 28 32 10.2176/nmc.39.28 10093457

[B7] Cheatham ML Block DF Nelson LD Evaluation of acute mental status change in the nonhead injured trauma patient Am Surg 1998 64 900 905 9731823

[B8] Harrop JS Sharan AD Vaccaro AR Przybylski GJ The cause of neurologic deterioration after acute cervical spinal cord injury Spine 2001 26 340 346 10.1097/00007632-200102150-00008 11224879

[B9] Prabhu V Kizer J Patil A Hellbusch L Taylon C Leibrock L Vertebrovasilar thrombosis associated with nonpenetrating cervical spine trauma J Trauma 1996 40 130 137 10.1097/00005373-199601000-00027 8576978

[B10] Carpenter S Injury of neck as cause of vertebral artery thrombosis J Neurosurg 1961 18 849 853 10.3171/jns.1961.18.6.0849 13876791

[B11] Lyness SS Simone FA Vascular complications of upper cervical spine injuries Orthop Clin North Am 1978 9 1029 1038 740371

[B12] Taylor MW Senkowski CK Bilateral Vertebral Artery Dissection after Blunt Cervical Trauma: Case Report and Review of the Literature J Trauma 2002 52 1186 1188 10.1097/00005373-200206000-00027 12045651

[B13] Marks RL Freed MM Nonpenetrating injuries of the neck and cerebrovascular accident Arch Neurol 1973 28 412 414 4701389 10.1001/archneur.1973.00490240072014

[B14] Schneider RC Crosby EC Vascular insufficiency of brain stem and spinal cord in spinal trauma Neurology 1972 19 312 354 10.1212/wnl.9.10.643 14443251

[B15] Friedman D Flanders A Thomas C Miller W Vertebral artery injury after acute cervical spine trauma AJR Am J Roentgenol 1995 162 443 447 10.2214/ajr.164.2.7839986 7839986

[B16] Kloen P Patterson JD Wintman BI Ozuna RM Brick GW Closed cervical spine trauma associated with bilateral vertebral artery injuries Arch Orthop Trauma Surg 1999 119 478 481 10.1007/s004020050027 10613246

[B17] Wirbel R Pistorius G Braun C Eichler A Mutschler W Bilateral vertebral artery lesion after dislocating cervical spine trauma. A case report Spine 1996 21 1375 1380 10.1097/00007632-199606010-00020 8725932

[B18] Louw JA Mafoyane NA Small B Neser CP Occlusion of the vertebral artery in cervical spine dislocations J Bone Joint Surg Br 1990 72 678 81 10.1302/0301-620X.72B4.2380226 2380226

[B19] Parent AD Harkey HL Touchstone DA Smith EE Smith RR Lateral cervical spine dislocation and vertebral artery injury Neurosurgery 1992 31 501 509 10.1227/00006123-199209000-00012 1407430

[B20] Inamasu J Guiot BH Vertebral artery injury after blunt cervical trauma: an update Surg Neurology 2006 65 238 246 10.1016/j.surneu.2005.06.043 16488240

[B21] Biffl WL Ray CE Jr Moore EE Mestek M Johnson JL Burch JM Noninvasive diagnosis of blunt cerrebrovascular injuries: a preliminary report J Trauma 2002 53 5 850 856 10.1097/00005373-200211000-00008 12435934

[B22] Hollingworth W Nathens AB Kanne JP Crandall ML Crummy TA Hallam DK Wang MC Jarvik JG The diagnostic accuracy of computed tomography angiography for traumatic or atherosclerotic lesions of the carotid and vertebral arteries: a systematic review Eur J Radiol 2003 48 88 102 10.1016/S0720-048X(03)00200-6 14511863

[B23] Bromberg WJ Collier BC Diebel LN Dwyer KM Holevar MR Jacobs DG Kurek SJ Schreiber MA Shapiro ML Vogel TR Blunt cerebrovascular injury practice management guidelines: the Eastern Association for the Surgery of Trauma J Trauma 2010 68 2 471 7 20154559 10.1097/TA.0b013e3181cb43da

[B24] Giacobetti FB Vaccaro AR Bos-Giacobetti MA Deeley DM Albert TJ Farmer JC Cotler JM Vertebral artery occlusion associated with cervical spine trauma. A prospective analysis Spine (Phila Pa 1976) 1997 22 2 188 192 10.1097/00007632-199701150-00011 9122799

[B25] Marshall LF Knowlton S Garfin SR Klauber MR Eisenberg HM Kopaniky D Miner ME Tabbador K Clifton GL Deterioration following spinal cord injury. A multicenter study J Neurosurg 1987 66 3 400 404 10.3171/jns.1987.66.3.0400 3819834

[B26] Hauswald M Ong G Tandberg D Omar Z Out-of-hospital spinal immobilization: its effect on neurologic injury Acad Emerg Med 1998 5 3 214 219 10.1111/j.1553-2712.1998.tb02615.x 9523928

